# High lifetime risk of cardiovascular disease vs low 10-year Framingham risk score in HIV-infected subjects under ART in Spain: the Coronator study

**DOI:** 10.7448/IAS.15.6.18109

**Published:** 2012-11-11

**Authors:** V Estrada, M Masiá, J Iribarren, C Sanchez-Marcos, F Lozano, C Miralles, J Olalla, J Santos, J Bernardino, F Dronda, P Domingo

**Affiliations:** 1 Hospital Clínico San Carlos, Madrid, Spain; 2 Hospital de ElcheElche, Spain; 3 Hospital Donostia, San Sebastián, Spain; 4 Hospital Virgen de Valme, Sevilla, Spain; 5 CHUVI-Xeral, Vigo, Spain; 6 Hospital Costa del Sol, Marbella, Spain; 7 Hospital Virgen de la Victoria, Málaga, Spain; 8 Hospital la Paz, Madrid, Spain; 9 Hospital Ramón y Cajal, Madrid, Spain; 10 Hospital Sant Pau, Barcelona, Spain

## Abstract

**Purpose:**

Due to the relative low age of HIV-infected patients, Framingham risk score (FRS) usually estimates a low CVD risk. Lifetime risk estimations use the risk of developing CVD over the course of an individual's remaining lifetime and may be useful in communicating the risk of CVD to young patients. Our aim is to estimate the lifetime risk of CVD in a representative sample of HIV patients under antiretroviral therapy in Spain.

**Methods:**

Cross-sectional analysis in 10 HIV units across Spain, including information on demographics, HIV disease status, treatment history and cardiovascular risk factors of subject under ART. Lifetime CVD risk was calculated with the method of Berry et al, which classifies the lifetime risk in five mutually exclusive categories: 1. All risk factors are optimal; 2. At least one risk factor is not optimal; 3. At least one risk factor is elevated; 4. One major risk factor is present; and 5. Two or more major risk factors are present. Risk factors included are cholesterol level, blood pressure, diabetes and tobacco smoking. We grouped these five categories in two major groups, low-risk (groups 1+2+3) and high-risk category (groups 4+5). We calculated the prevalence of having a high lifetime risk, and its crude and aOR (adjusted by age, sex, place of origin, education level, transmission category, time since HIV diagnosis, CDC stage, current and nadir CD4 count, HCV coinfection, time on current and total ART, being on the first ART regimen, and PI vs. NNRTI regimen).

**Results:**

We included 839 subjects free of previous CVD disease: 72% men, median age 45.6y, median CD4 count 598 cells, median time since HIV diagnosis 11y, median time on ART 6.3y, 87% had undetectable VL. Estimated 10-year CVD risk was low (<5%) in 78% of the patients, and intermediate (5–10%) in 20%. Lifetime risk estimation shows a high risk profile for 71.4% of the population studied (≥1 major risk factors). Factors significantly and independently associated with an increased lifetime risk were older age, non-Spanish origin and longer time on ART. Adjusted OR for patients on ART longer than 10 years (vs<5 years) was 2.2 [95% CI 1.13–4.34]. No relationship was found with current or nadir CD4 lymphocyte counts, CDC stage C, HCV confection or type of ART.

**Conclusions:**

There are significant disparities between the low 10y CVD risk estimated with FRS and the elevated lifetime risk in HIV patients on ART. Prolonged ART is associated with an increased CVD lifetime risk.
